# Pleural Effusion: A Rare Side Effect of Nilotinib—A Case Report

**DOI:** 10.1155/2014/203939

**Published:** 2014-09-09

**Authors:** Hava Üsküdar Teke, Olga Meltem Akay, Deniz Gören Şahin, Mustafa Karagülle, Eren Gündüz, Neslihan Andıç

**Affiliations:** Hematology, Medical Faculty, Eskişehir Osmangazi University, 26090 Eskişehir, Turkey

## Abstract

Pleural effusion, as a side effect of tyrosine kinases, may be seen as most commonly associated with dasatinib and very rarely seen with nilotinib. In this report we present a chronic phase of CML case that was treated with nilotinib due to imatinib (Gleevec) allergy and had pleural effusion with nilotinib at 5th year of treatment. If pleural effusion develops in patients taking nilotinib and if this effusion is exudative and lymphocyte predominant, after ruling out pulmonary and cardiac etiologies, it must be associated with nilotinib; according to stage of effusion drug should be discontinued and/or steroid should be started and/or surgery should be performed.

## 1. Introduction

Chronic myeloid leukemia (CML) is a chronic myeloproliferative disease characterized by tyrosine kinase activity caused by translocation between chromosomes 9 and 22 [1]. Oral medications for CML include tyrosine kinase inhibitors as imatinib, dasatinib, and nilotinib. The incidence of dasatinib related pleural effusion is about 7–35% [2]. While pleural effusions are seen less often with imatinib, those seen with nilotinib are very rare (<1%) [3].

In this report, we present a chronic phase of CML case that was treated with nilotinib due to imatinib (Gleevec) allergy and had pleural effusion with nilotinib at 5th year of treatment.

## 2. Case

**Figure 1:**
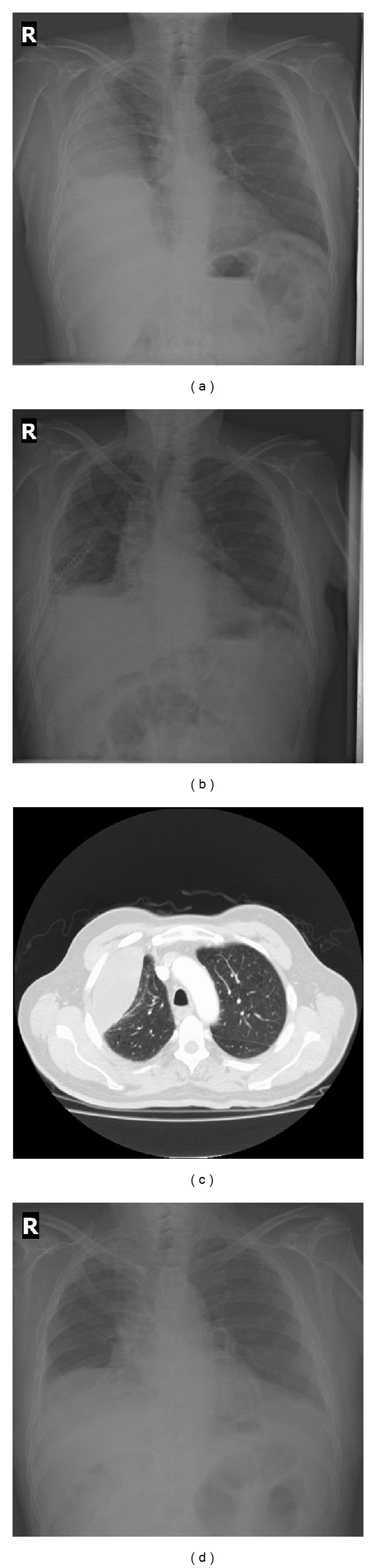
Posteroanterior chest X-ray in admission (a), after catheter thoracostomy (b) and thorax tomography (c), and posteroanterior chest X-ray after steroid treatment (d).

A 68-year-old male patient presented to emergency department with complaints of high fever, in April 2008. The only pathological finding in physical examination was presence of palpable spleen 2 cm below costal margin. Blood analysis showed Hb 11 g/dL, hematocrit 30.2%, white blood cell count 126800/mm^3^, absolute neutrophil count (ANC) 93800/mm^3^, platelets 672000/mm^3^, and C-reactive protein 1.15 mg/dL (0 to 0.8). In biochemical analysis, lactate dehydrogenase (LDH) was found as 1164 U/L apart from other biochemical parameters, which were within normal limits. In peripheral blood smear immature granulocytes, basophilia and eosinophilia were observed. In bone marrow aspiration, increased myeloid cells and myeloid/erythroid ratio of 50–60/1 were found. Cytogenetic and PCR analyses showed the presence of Philadelphia chromosome. Calculated Sokal risk score was 0.98 (intermediate). Imatinib mesylate 400 mg/day was started. After 1 month of Gleevec treatment, patient presented with swelling and burning sensation in his eyes and itchy red lesions on arms and legs. Imatinib mesylate treatment was discontinued due to skin rashes and dexamethasone treatment was started. Lesions disappeared and treatment was continued with nilotinib. Patient was free of Ph chromosome in cytogenetic analysis of bone marrow and had major molecular response in PCR during nilotinib treatment. Then he admitted with dyspnea limiting daily activities. Physical examination revealed normal vital findings, O_2_ saturation of 95%, and absence of respiratory sounds at basal and middle zones of right lung. The complete blood count showed Hb of 11.4 g/dL, WBC of 6.7 × 10^3^/*μ*L, and platelet count of 327 × 10^3^/*μ*L. Presence of pleural effusion was found in chest X-ray (Figure 1(a)). The patient was hospitalized and nilotinib treatment was discontinued. Viral markers for hepatitis, cytomegalovirus, and HIV were negative. Catheter thoracostomy was performed (Figure 1(b)). Analysis of pleural fluid was exudative and lymphocyte predominant (400/*μ*L [98%]). Cytology of pleural fluid was also negative for malignancy. Thorax tomography was performed (Figure 1(c)) and pulmonary thromboembolism was ruled out. Because of the exudative effusion, findings of a suspicious mass appearance in chest X-ray, and pleural thickening in thorax tomography, he was operated on by thoracic surgery with preliminary diagnosis of pulmonary malignancy. Total decortication and intercostal blockage were performed. Cytology and pathology results were negative for malignancy. After exclusion of pulmonary and cardiac etiologies, clinic picture of the patient was attributed to nilotinib and treatment with diuretic and methylprednisolone 32 mg/day was started. Approximately 1.5–2 months after steroid treatment pleural effusion almost completely regressed, nilotinib 2 × 200 mg/day was started again and steroid treatment was gradually discontinued. After complete disappearance of pleural effusion (Figure 1(d)) nilotinib dose was increased to 2 × 400 mg/day.

## 3. Discussion

2nd generation tyrosine kinase inhibitors, dasatinib and nilotinib, were used in the treatment of imatinib intolerant or resistant CML patients [4]. In our patient due to imatinib related skin reactions, he was regarded as imatinib intolerant and treatment was switched to nilotinib. The frequency of dasatinib related pleural effusion is more than that of nilotinib. Dasatinib related pleural effusion risk was found increased in CML patients with previous cardiac problems and hypertension and receiving dasatinib twice daily instead of one [5]. Although the mechanism of dasatinib related pleural effusion is not fully understood, it is thought to be associated with PDGFR*β* inhibition [6]. Nilotinib is a second generation tyrosine kinase inhibitor, inhibiting KIT and PDGFR besides ABL. It is 30 times more potent than imatinib to Bcr-Abl. When nilotinib is compared with imatinib and dasatinib, its selectivity to ABL is more than that to KIT and PDGFR. Its significantly low selectivity to PDGFR explains why nilotinib related pleural effusion is seen less than 1% [4, 7]. Analysis of clinical studies suggests that cross-intolerance to nilotinib is rare in imatinib intolerant patients [4]. Our patient is one of the rare cases that developed nilotinib related pleural effusion. Because of imatinib intolerance presenting with skin reactions, his treatment had been switched to nilotinib. He developed nilotinib related pleural effusion after 5 years of treatment.

In case of pleural effusion caused by tyrosine kinase inhibitors, treatment should be performed according to degree of effusion. When an asymptomatic patient with effusion is detected, they must be closely monitored without interrupting treatment [8]. If grade 2 or 3 symptomatic pleural effusion is present, discontinuing tyrosine kinase inhibitors may provide a response to treatment. If symptoms are severe, resolution can be achieved in 72 hours with prednisone 40 mg/day [5]. Over grade 3, if severe shortness of breath is present, usually therapeutic thoracentesis, catheter thoracostomy, and pleuroperitoneal shunt may be required [6]. Since our patient was grade 3 and had pleural effusion extending to middle zone of right lung and preventing daily activities, catheter thoracostomy was performed. In our patient, pulmonary thromboembolism, malignancy, and cardiac etiology were ruled out and right-sided lymphocyte predominant exudative effusion was considered as a side effect of nilotinib. Bergeron et al. reported [9] a case series of dasatinib related lung abnormalities and showed that four out of nine patients had right-sided pleural effusion the same as in our case. In analogy to the management of dasatinib, related pleural effusion prednisolone 32 mg/day was started to the patient whose treatment was discontinued for nilotinib related pleural effusion. However, in our patient significant reduction and/or disappearance of pleural effusion occurred weeks later rather than days as in dasatinib related pleural effusion treatment. After significant decrease of effusion, nilotinib was started again with low dose of 400 mg/day; then dose was increased to 800 mg/day and during this period recurrence of pleural effusion was not observed.

## 4. Conclusion

Pleural effusion, as a side effect of tyrosine kinases, may be seen as most commonly associated with dasatinib and very rarely seen with nilotinib. If pleural effusion develops in patients taking nilotinib and if this effusion is exudative and lymphocyte predominant, after ruling out pulmonary and cardiac etiologies, it must be associated with nilotinib; according to stage of effusion, drug should be discontinued and/or steroid should be started and/or surgery should be performed.
